# Real-time detection of carboplatin using a microfluidic system†
†Electronic supplementary information (ESI) available. See DOI: 10.1039/C6AN01446A



**DOI:** 10.1039/c6an01446a

**Published:** 2016-08-26

**Authors:** Tonghathai Phairatana, Chi Leng Leong, Sally A. N. Gowers, Bhavik Anil Patel, Martyn G. Boutelle

**Affiliations:** a Institute of Biomedical Engineering , Faculty of Medicine , Prince of Songkla University , Songkhla , Thailand . Email: t.phairatana@gmail.com; b Department of Bioengineering , Faculty of Engineering , Imperial College London , London , UK . Email: m.boutelle@imperial.ac.uk; c School of Pharmacy and Biomolecular Sciences , University of Brighton , East Sussex , UK

## Abstract

A microfluidic sensor system based on a carbon nanotube-epoxy composite electrode was fabricated to allow detection of the presence of the anti-cancer drug carboplatin in healthy tissue in real time during chemotherapy. Detection of carboplatin was carried out by observing the effects of the drug on the differential pulse voltammetry of free purine bases using a novel carbon nanotube-epoxy composite electrode. In free solution these electrodes performed better than glassy carbon electrodes for oxidation of the free purine bases AMP and GMP, and than DNA-modified carbon nanotube-epoxy composite sensors for detection of carboplatin. On-line carboplatin detection was performed using a computer-controlled microfluidic platform. The methodology for on-line carboplatin detection was optimised in terms of the analysis time and to allow repeated carboplatin measurement using the same electrode. Microdialysis sampling and our microfluidic platform were combined to give a proof-of-concept system for real-time carboplatin detection with a limit of detection of 0.014 μM carboplatin in the sampled media. This paper is dedicated to Craig Lunte's pioneering work in analysis and microdialysis.

## Abstract 2

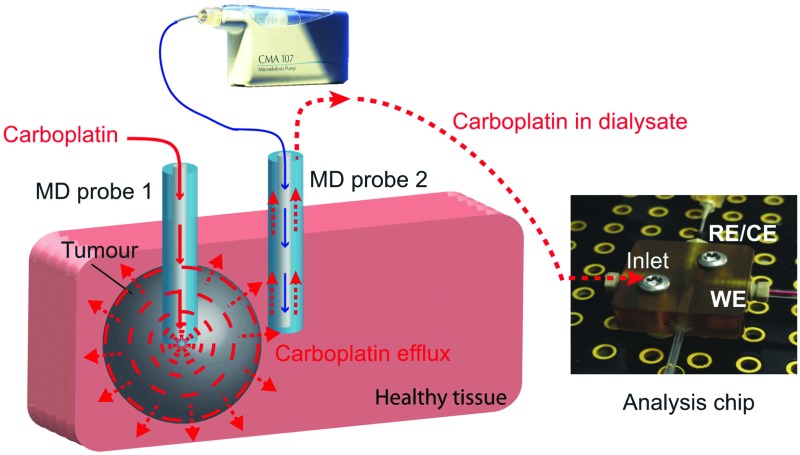
A novel on-line microfluidic assay for the chemotherapy agent carboplatin will allow sensitive detection of the drug directly applied to the tumour as it emerges from the tumour into healthy tissue.

## 1 Introduction

Chemotherapy is central to most cancer treatment regimes. It usually involves systemic administration of one or more anti-cancer drugs, which are designed to kill cancer cells and hence inhibit the growth of tumours in order to prevent their spread to other organs. Platinum-based anti-cancer drugs such as oxaliplatin, nedaplatin, lobaplatin, heptaplatin and carboplatin are used for chemotherapy.^1^ Among these drugs, carboplatin is most widely used for the treatment of a variety of malignancies. Carboplatin has proven activity against a variety of solid tumours including ovarian, testicular, head and neck and lung cancers (both non-small-cell and small-cell).^2–6^ However, although carboplatin is an effective anti-cancer drug, it causes many side effects. This is because, in common with all anti-cancer drugs, carboplatin works by binding to the DNA of active cells that are growing and dividing and as a result it cannot distinguish between cancer cells and healthy cells such as blood cells, skin cells, hair follicles and the cells inside the digestive system. Consequently, carboplatin affects not only the cancer cell growth but also blocks the growth of healthy rapidly dividing cells, leading to side effects such as mouth and throat sores, diarrhea, nausea and vomiting, blood disorders, nervous system effects, appetite loss and hair loss.^7,8^

Several analytical techniques have been used to detect the interaction of carboplatin with the structure of deoxyribonucleic acid (DNA). These include high-performance liquid chromatography, inductively coupled plasma-mass spectrometry and capillary electrophoresis.^9–13^ These methods involve large instruments with high costs, require highly-trained personnel for testing and as they are sample-based give poor time resolution. There is a need for rapid, continuous, low-cost techniques for analysis of drug–DNA interaction and high time resolution. Electrochemical techniques have high sensitivity, low cost, and rapid response. Typically once the method is established they do not require a great deal of user expertise. This has made electrochemical devices an attractive choice for point-of-care applications with high time resolution.

Carboplatin is not electroactive and therefore cannot be detected directly using electrochemistry, however, it can be detected through its effect on DNA bases, which are electrochemically active. These interactions have been studied with a range of electrodes including hanging mercury drop electrodes, screen printed electrodes, graphite electrodes and glassy carbon electrodes.^14–17^ Carboplatin has been detected electrochemically through its effects on DNA electrochemistry by the Brett-Oliveira group using glassy carbon electrode (GCE).^17^ Initially, the GCE was coated with DNA to detect the oxidation peaks due to four DNA bases (adenine, guanine, cytosine and thymine),^18^ of these only guanine and adenine respond to carboplatin. It was also necessary to condition the DNA sensors in single-stranded (ss) DNA (forming a triplex structure) to stabilise DNA-base oxidation peaks.^19^

The problem with carboplatin is that its therapeutic index (the ratio between effective dose and toxic dose) is low. Consequently, avoiding toxicity by controlling the systemic dose is difficult. Recently, there has been a move to bioengineer devices that allow personalisation of cancer treatment by applying the drugs either singly or in combination directly to the tissue.^20^ At present these approaches use histology to determine drug effectiveness followed potentially by systemic administration of the drugs.^21,22^ An alternative approach is to consider treatment by delivering the drug directly to a targeted specific area (the tumour) at a high dose and to monitor the surrounding healthy tissue for the first arrival of the drug. This could be achieved either by detecting carboplatin or DNA damage caused by carboplatin in real time. Such an approach could enhance the effectiveness of chemotherapy, would reduce side effects and would also reduce the cost of drug administration as the drug would be injected directly into the cancer cells.

Microdialysis (MD) is a powerful sampling technique that is capable of continuously monitoring the extracellular space both *in vivo* and *in vitro*. MD sampling has been used extensively for monitoring amino acids, neurotransmitters and energy metabolites such as glucose, lactate and pyruvate in the brain for both fundamental and clinical research studies.^23–25^ Microfluidics has attracted a great deal of attention for miniaturisation of diagnostic devices due to their low analysis volume, short time for analysis, compact size and low cost^26^ and is particularly well suited for analysis of the low-volume flow streams produced with microdialysis.

In addition to monitoring analytes, sampling probes have also been used to deliver drugs or other substances for pharmacokinetic and drug metabolism studies in the blood and various tissues of both animals and humans, and have also been used to investigate drug distribution to specific regions.^27,28^ Retrodialysis using a microdialysis probe could allow clinicians to deliver carboplatin directly to the tumour site.

**Fig. 1:**
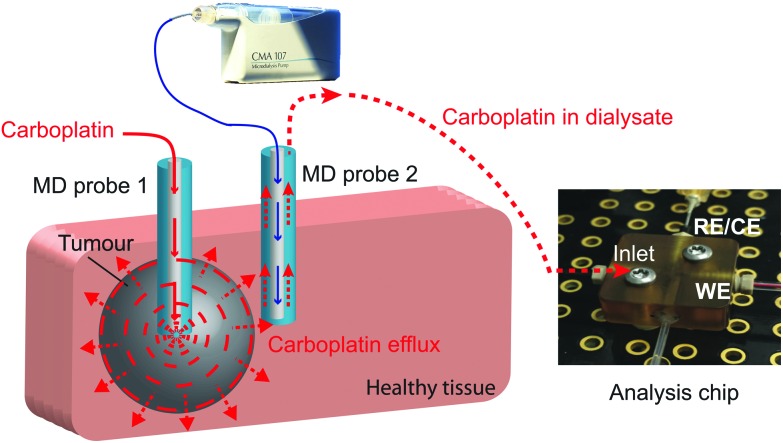
Schematic of an on-line carboplatin detector in healthy tissue during chemotherapy.

In this study we present a novel microfluidic sensing system that we envisage being used alongside retrodialysis drug delivery into the tumour to detect in real time when carboplatin disperses into surrounding healthy tissue. The concept is shown in Fig. 1. Development of the detection system involved investigation of the interaction between carboplatin and DNA nucleotides (guanine and adenine) by electrochemical detection of DNA damage using both a free-solution and on-chip setup. We have used a novel multiwalled carbon nanotube (MWCNT)-based composite electrode coupled to differential pulse voltammetry (DPV), which is suitable for working in the low-volume, high-salt microfluidic environment.

## 2 Materials and methods

### 2.1 Materials and reagents

Carboplatin (Sigma-Aldrich) was prepared in 0.1 M acetate buffer (ACB, pH 4.5). T1 perfusate solution was prepared with 147 mM sodium chloride, 4 mM potassium chloride, and 2.3 mM calcium chloride in distilled water and 400 μl of 5-chloro-2-methyl-4-isothiazoline-3-one (Kathon CG, SUPELCO Analytical, USA) was added as an antibacterial agent. All chemicals were of analytical grade and obtained from Sigma-Aldrich. Guanosine 5-monophosphate disodium salt hydrate (GMP, from yeast, 99%) and adenosine 5-monophosphate (AMP, from yeast, sodium 99%, premium grade) were obtained from Sigma-Aldrich. Stock solutions of both 1 mM GMP and 1 mM AMP were dissolved in ACB pH 4.5 as a supporting electrolyte for use in a beaker experiment. In the case of testing *in vitro* using an autocalibration system, 1.5 mM AMP was dissolved in 0.1 M ACB and T1 solution mixed in the ratio of 3 : 1.

### 2.2 MWCNT-epoxy composite fabrication

Multiwalled carbon nanotubes (MWCNT, 6–13 nm OD, 2.5–20 μm length) were purchased from Sigma-Aldrich. Epoxy resin and hardener were obtained from Robnor (RX771C/NC, and HY1300). The method of MWCNT-epoxy composite fabrication is reported previously by the Patel group,^29^ although in the present work we use a different ratio of MWCNT : epoxy. Briefly, plastic pipette tips (10 μl size) were employed to contain the carbon-epoxy composite. Epoxy resin was prepared by mixing resin and hardener in the ratio of 3 : 1. The MWCNT were thoroughly combined with the epoxy resin in the proportion of 1 : 4 by weight and stirred well. A pipette tip was filled densely with the MWCNT-epoxy composite paste by a tapping and dragging method. Then an electrical wire was inserted into the filled pipette and it was allowed to dry at room temperature for two days. The epoxy resin with the same ratio as mentioned above was used to fill up the internal volume of the pipette tip using a 18G hypodermic needle, and it was allowed to cure for two days. After this process, a scalpel was used to cut the end of the electrode to expose a surface area of the MWCNT-epoxy composite that was approximately 0.50 mm in diameter. In the microfluidic platform experiment, MWCNT-epoxy composite electrodes were fabricated using Radel™ tubing (0.50 mm ID, IDEX) instead of the pipette tip.

### 2.3 Electrochemical and microfluidic instrumentation

Preliminary experiments were conducted in a small glass beaker using the MWCNT-epoxy composite electrode as a working electrode. A coiled Pt wire and a commercial Ag|AgCl in 3 M NaCl solution (BASi) were used as a counter and a reference electrode, respectively.

In this work, differential pulse voltammetry (DPV) was carried out using Echem software (eDAQ, UK) and a PowerLab 8SP (ADinstruments). Data was processed using Igor Pro software.

**Fig. 2:**
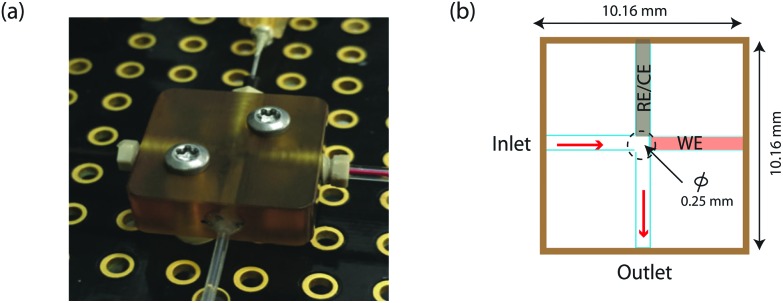
On-line analysis chip (a) photograph of the on-line analysis chip based on Labsmith TEE component. (b) Schematic of the analysis chip showing the positions of a combined reference and counter needle electrode (RE/CE) and a working electrode (WE).

A novel on-line analysis chip was fabricated based on a microfludic interconnect TEE (Labsmith Inc, USA). A new channel was drilled to make a cross shape for the outlet channel and a piece of tube was glued to connect the channel using Araldite glue. This is shown schematically in Fig. 2. We estimate the volume between the electrodes to be approximately 2.6 nanolitres.

## 3 Results and discussion

### 3.1 Free-solution experiments

#### 3.1.1 Response of MWCNT-epoxy composite electrode to GMP and AMP

**Fig. 3:**
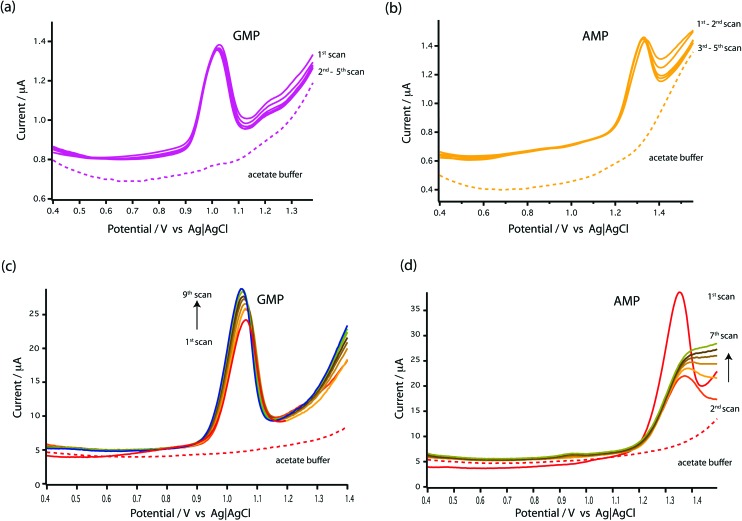
DPV voltammograms of stability study at a bare MWCNT-epoxy composite electrode in (a) 1 mM GMP and (b) 1 mM AMP as compared with at a bare GCE in (c) 1 mM GMP and (d) 1 mM AMP. DPV was performed at a scan rate of 5 mV s^–1^, pulse amplitude of 50 mV and pulse width of 70 ms. The dotted line indicates as DPV voltammograms obtained using 0.1 M acetate buffer at pH 4.5 as a supporting electrolyte.

The electrochemical response of MWCNT-epoxy composite pipette tip to 1 mM GMP and 1 mM AMP using differential pulse voltammetry (DPV) is shown in Fig. 3a and b for 5 DPV scans. For comparison Fig. 3c and d show the results for GMP and AMP at a bare GCE.

As seen in Fig. 3c and d, the results showed that the oxidation peaks of both nucleotides (GMP and AMP) were not stable or reproducible with a GCE. For AMP, the oxidation peak obtained at the GCE diminished with successive scans (see Fig. 3d). This agrees with the finding of Piedade^19^ and is the reason why Oliveira-Brett's group modified the glassy carbon electrode with DNA followed by conditioning with ssDNA.

Surprisingly, stable oxidation currents were observed within a few DPV scans at the bare MWCNT-epoxy composite electrode for both AMP and GMP solutions. These DPV voltammograms show prominent oxidation peaks at +1.0 V for GMP (Fig. 3a) and at +1.30 V for AMP (Fig. 3b). These oxidation peaks were at almost the same potentials as observed for the DNA-modified electrode.^19^

The MWCNT-epoxy composite electrodes gave more stable oxidation peaks than at GCEs, probably because composite electrodes provide improved mass transfer and enhanced electrochemical behaviour.^30^ There is much in the recent literature on the advantages of graphene, boron-doped diamond and carbon nanotube-based electrodes including their fast electrochemistry, chemical stability and resistance to fouling.^31^ Indeed we have recently built an ultra-sensitive flow-cell for dopamine detection based on metallic single-walled carbon nanotubes.^32^ Our choice of a MWCNT-epoxy composite electrode was a compromise between excellent electrochemical performance and ease of fabrication into an electrode small enough to be incorporated into a robust microfluidic cell.

#### 3.1.2 Determination of carboplatin

**Fig. 4:**
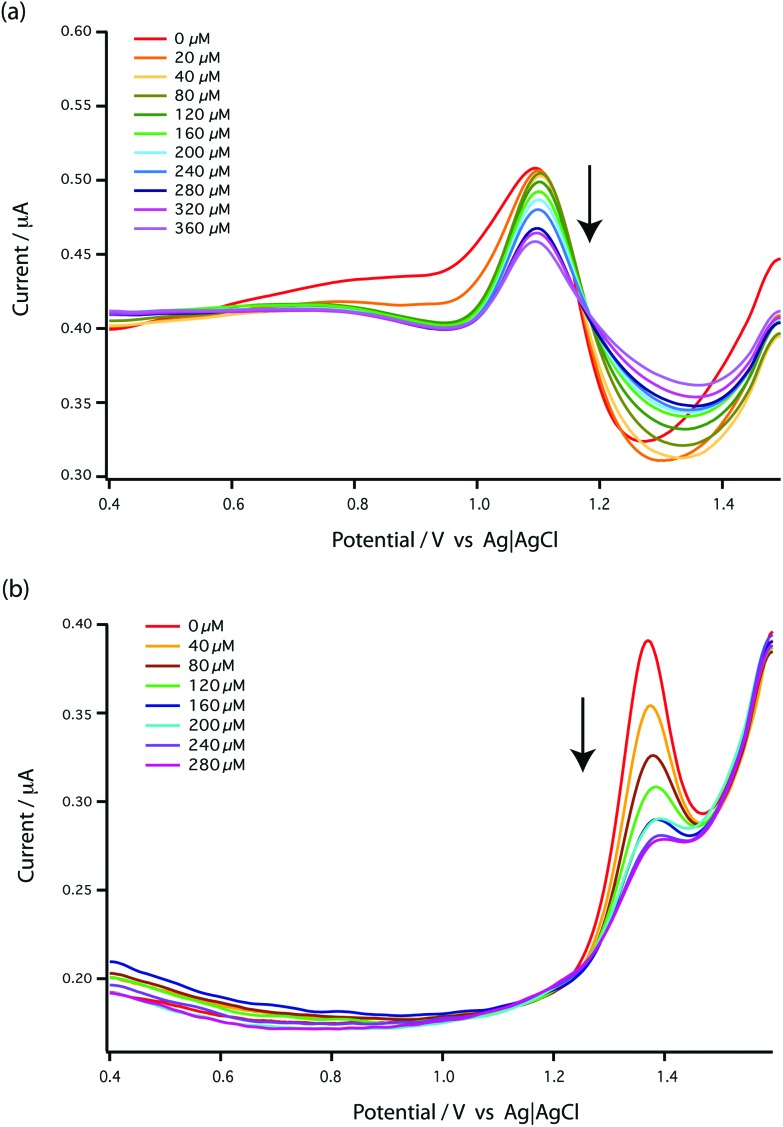
DPV voltammograms of bare MWCNT-epoxy composite electrodes for the addition of carboplatin to (a) 1 mM GMP and (b) 1 mM AMP in 0.1 M acetate buffer pH 4.5 as a supporting electrolyte at a scan rate of 5 mV s^–1^, pulse amplitude of 50 mV and pulse width of 70 ms.

**Fig. 5:**
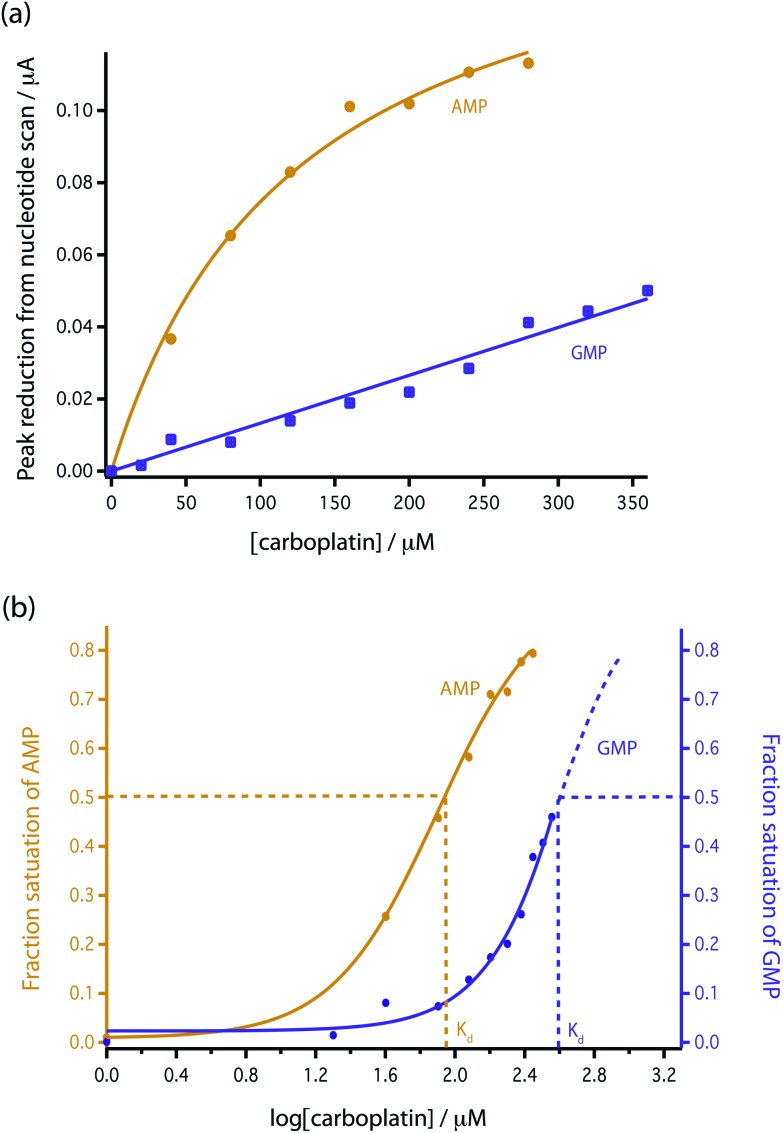
Comparison of the reduction in peak height with addition of carboplatin for GMP and AMP using bare MWCNT-epoxy composite electrodes. (a) Data fitted according to eqn (4). (b) Fractional binding of DNA bases to carboplatin *vs.* log[carboplatin] μM^–1^.

As bare MWCNT-epoxy composite electrodes showed such stable electrochemistry for the free nucleotides we investigated the effect of different carboplatin concentrations directly on the signals of free GMP and AMP. The results are shown in Fig. 4. As can be seen, both GMP and AMP show a clear dose dependency on carboplatin. For GMP (Fig. 4a) the effect is on both peak and post peak areas and the use of a baseline between pre- and post-peak troughs reveals a fall in peak height. The reason for this complex behaviour is not clear. For AMP (Fig. 4b) a clear fall in peak height is seen. In Fig. 5 the reduction in peak height is shown as a function of carboplatin concentration on both linear (Fig. 5a) and semi-log (Fig. 5b) scales. In Fig. 5a we find that the AMP peak is more sensitive to carboplatin concentration showing some apparent saturation at higher concentrations.

To model this behaviour we can consider *K*_d_, which is the dissociation constant. A reversible binding equilibrium is shown in eqn (1). In this case, the binding reaction of carboplatin (CP) to AMP is shown below:

1 AMP + CP ⇌ AMP{·}CP

For the above reaction, *K*_d_ is given by eqn (2).2
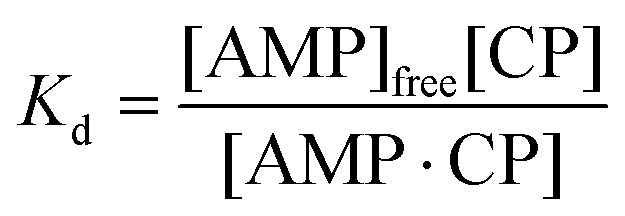



By balancing the mass of AMP:

3 [AMP]_0_ = [AMP]_free_ + [AMP{·}CP]

Substituting [AMP]_free_ by [AMP]_0_ – [AMP·CP], the equation for this case is given in eqn (4)4
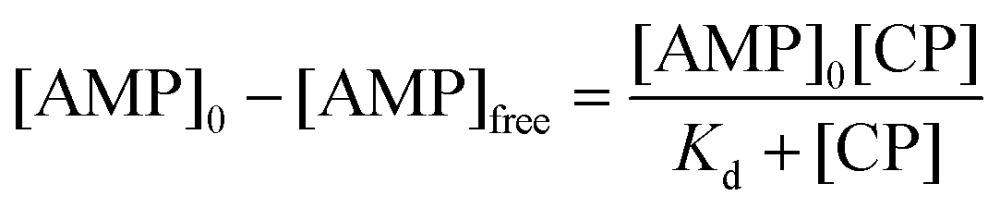



This [AMP]_0_ – [AMP]_free_ term corresponds to the loss of peak height (reduction of oxidation current). The fitted lines for the GMP and AMP results are shown in Fig. 5a. A value of *K*_d_ for carboplatin interacting with AMP was 124.9 ± 17.3 μM. For GMP, saturation was not reached with the carboplatin concentrations used, so it is more difficult to accurately determine *K*_d_. Nevertheless we can estimate *K*_d_ of GMP to be approximately 398 ± 20 μM. It is quite useful here to plot the data on a semi-log graph to give the curves shown in Fig. 5b. If we make the assumption that the interaction between the DNA bases and carboplatin is a simple binding event then *K*_d_ is when log[carboplatin] is a half. The data in Fig. 5b show that the sigmoid curve of GMP (dark blue) is shifted to the right reflecting the higher *K*_d_ (lower affinity for carboplatin) compared to AMP. Broadly similar results were found by Oliveira-Brett *et al.*, although they found the response saturated at 80 μM carboplatin in the same concentration of AMP using a DNA-modified GCE.^17^

We chose AMP for the on-line experiments mainly due to its greater sensitivity in the 0–100 μM range (Fig. 5). If carboplatin concentrations greater than 200 μM had been required, GMP would have been the preferred substrate. A second advantage of AMP is its simpler electrochemistry as shown in Fig. 4. Although it is not clear why, the baseline before and after the oxidation peak of AMP was found to be more stable than for GMP, as shown in Fig. 4a and b, allowing easier determination of low concentrations of carboplatin.

### 3.2 On-chip experimental setup

To realise the on-line analysis capabilities required for the schematic shown in Fig. 1, we set up a microfluidic-based system using the same MWCNT-epoxy composite electrode in a custom-made flow cell (Fig. 2). A combined needle electrode was used for the counter (stainless steel of the hypodermic needle) and the reference electrode (50 μm diameter Ag|AgCl wire). The rest of the analysis system was built using Labsmith computer-controlled valves and syringe pumps.

Whilst the DNA-base electrochemistry is stable (Fig. 3) and addition of carboplatin causes the size of the oxidation peak to decrease (Fig. 4), the latter effect was found to be irreversible. As a result it was necessary to carefully optimise the analysis procedure for continuous on-chip detection. We found that holding the electrode at a negative potential was sufficient to regenerate its electrochemical activity, presumably as a result of mild electrochemical cleaning.

**Fig. 6:**
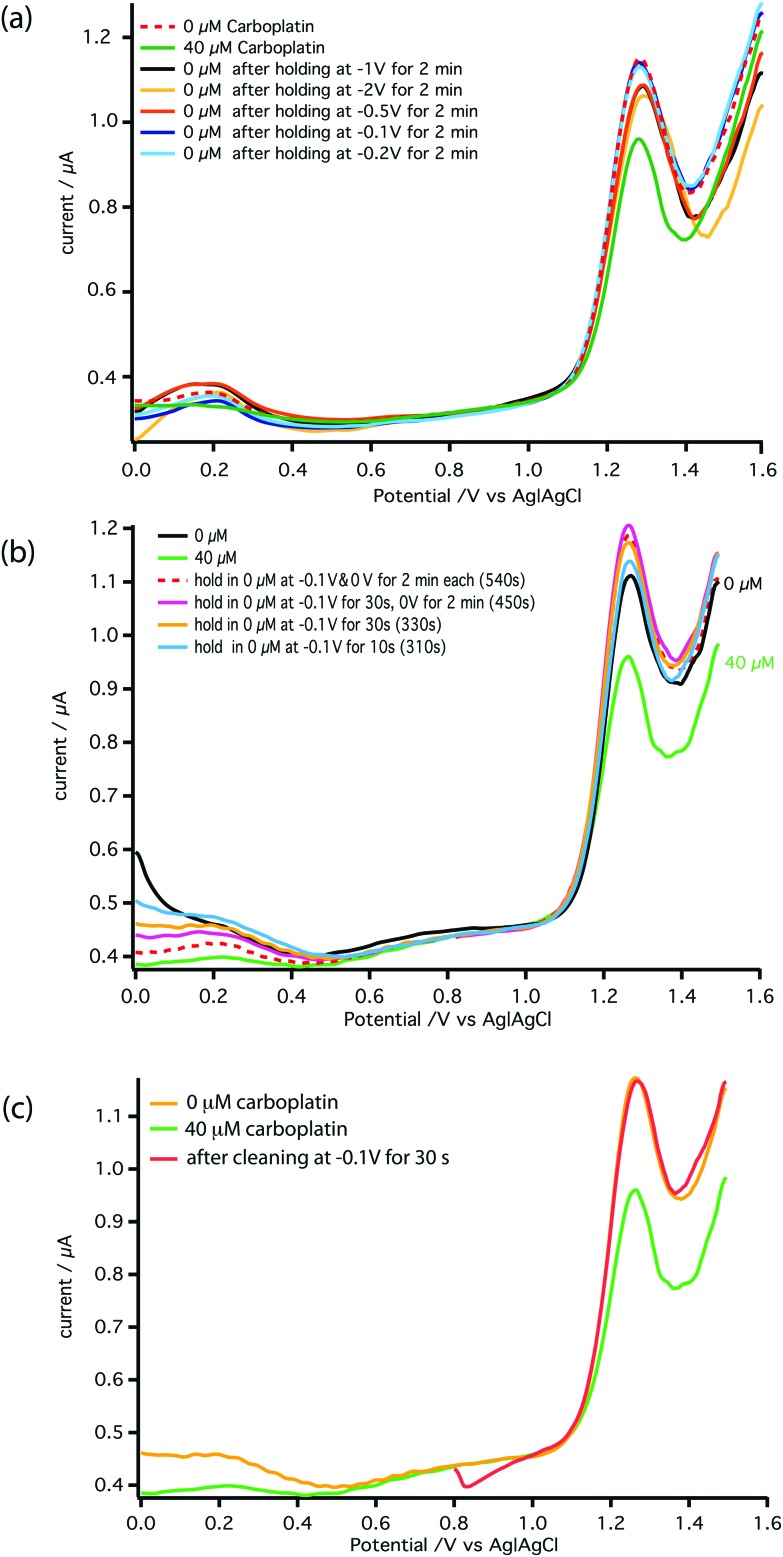
Optimisation of the conditions for detection of carboplatin–AMP interaction at MWCNT-epoxy composite electrodes. (a) The electrode was held at a negative potential for 2 min between scans to attempt to regenerate its electrochemical activity. Holding potentials of –0.1 V, –0.2 V, –0.5 V, –1 V and –2 V were tested. (b) The time that the electrode was held at –0.1 V and then at 0 V was varied to ascertain the minimum length of time required at each stage. (c) The effect of shortening the DPV scan range was tested to reduce the time required for each scan. All scans were carried out with a pulse amplitude of 50 mV, a pulse width of 70 ms and a scan rate of 5 mV s^–1^.

**Fig. 7:**
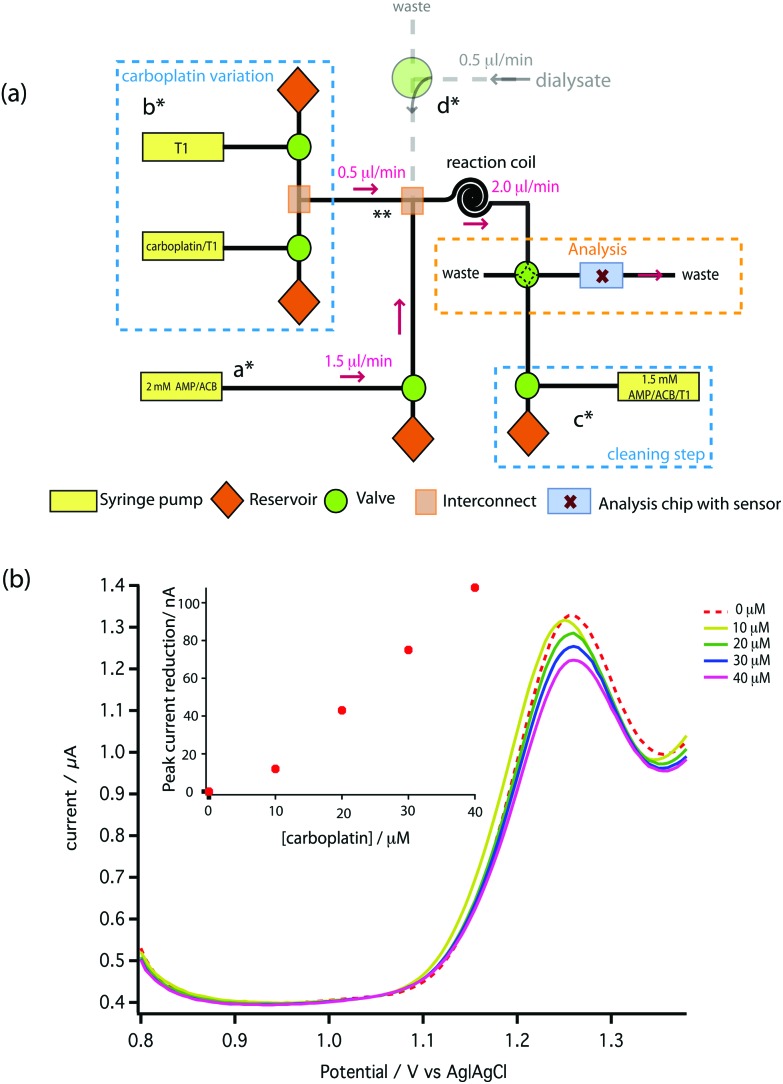
(a) Schematic of the microfluidic platform for carboplatin calibration. (b) The result of on-chip carboplatin calibration showing DPV voltammograms performed with a pulse amplitude of 50 mV, a pulse width of 70 ms and a scan rate of 5 mV s^–1^.

Fig. 6a shows the effect of the inter-scan holding potential on recovery of the electrochemical activity. We find –0.1 V (Ag|AgCl) provides the best compromise between signal recovery and maintenance of scan baseline. Addition of this step, however, increased the time between DPV scans lowering temporal resolution. Therefore, we optimised the duration of the –0.1 V step, as shown in Fig. 6b and the DPV voltage scan range as shown in Fig. 6c. The result is a stable measurement of carboplatin concentration every 170 s. Full details are described in the ESI.† The schematic of the microfluidic platform for carboplatin calibration is shown in Fig. 7a.

To carry out initial testing on the board, AMP was supplied to the analysis chip directly from a syringe pump (a*). Carboplatin dissolved in T1 solution at any concentration between zero and the syringe concentration of 160 μM is provided by the careful variation of flow rates of syringes and control of valves shown in b*. The two solutions mixed at point ** and were allowed to react for 2 min at 2 μl min^–1^ using a reaction coil before feeding into the analysis chip. In Fig. 7b the red dotted line shows the DPV scan for 1.5 mM AMP (on-chip concentration), together with the scans following mixing of increasing doses of carboplatin. Pleasingly, a clear dose-dependent reduction of the AMP oxidation peak is seen on-chip, which is approximately linear at these low concentrations compared to our measured *K*_d_. We can clearly resolve 10 μM carboplatin, and based on the SD of the AMP oxidation peaks we estimate the limit of detection is 0.49 μM.

### 3.3 Microdialysis experiment

The microfluidic system was coupled with an MD probe in order to validate the capability of detecting carboplatin physiologically during cancer chemotherapy. In terms of the schematic shown in Fig. 7a, AMP is supplied from syringe pump a*, but the carboplatin contained in position b* was not used. Instead of the carboplatin at b*, the MD stream at point d* reacts at the mixing point **.

**Fig. 8:**
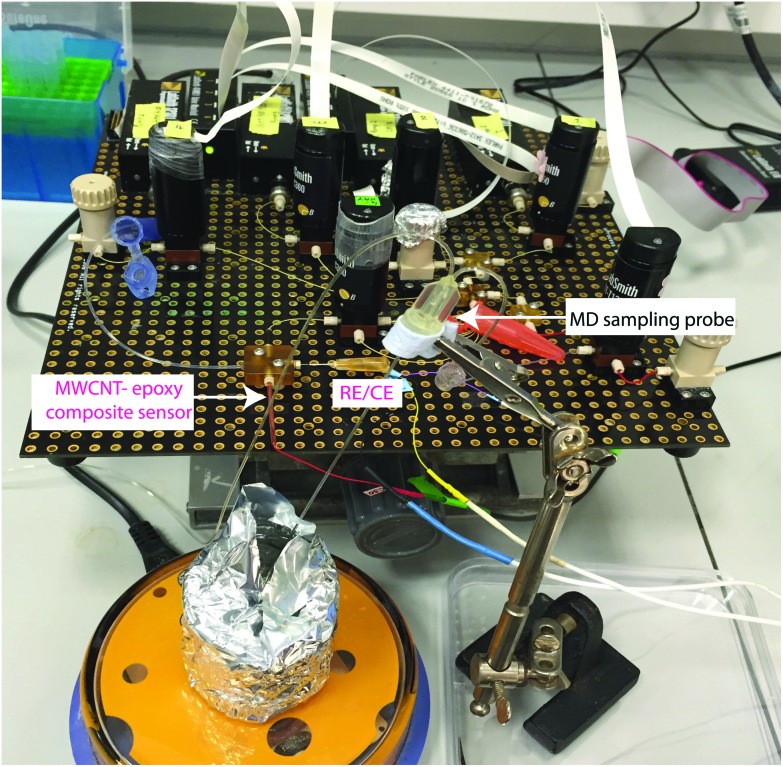
Photograph of the experimental setup using the microfluidic platform coupled with an MD sampling probe for carboplatin detection in real time.

A photograph of the complete system is shown in Fig. 8. An MD probe (CMA 70, MDialysis, Sweden) was immersed in a beaker containing 30 ml of T1 solution under stirred conditions. The outlet of the MD probe was connected to the microfluidic platform and the inlet of the MD probe was continuously perfused with T1 solution at 0.5 μl min^–1^ flow rate using a portable syringe pump (CMA 107, Mdialysis, Sweden). The T1 solution from the syringe pump flowed continuously during the experiment in order to allow carboplatin molecules to diffuse through the membrane and to travel in the liquid outlet stream towards the analysis system. The dialysate interacted with AMP in the microfluidic system and flowed towards the analysis chip for detection at the WE.

For the dialysate analysis, DPV voltammograms were obtained when the MD probe was placed in a well-stirred beaker containing T1 solution. Aliquots of 1 mM carboplatin standard solution were added to the beaker to give final concentrations of 0, 10, 20 and 25 μM.

**Fig. 9:**
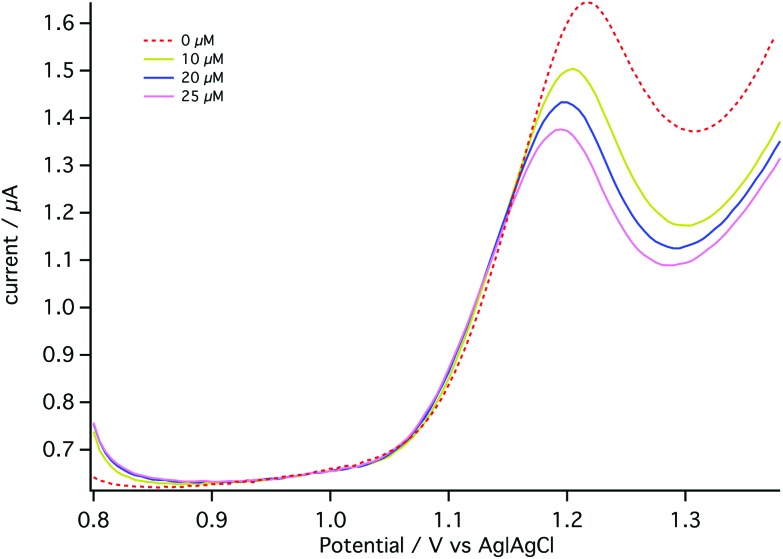
MD data using the autocalibration board. DPV voltammograms were obtained when aliquots of carboplatin standards were added into the beaker in which the MD probe was placed and the dialysate interacted with the 1.5 mM AMP/T1/ACB on-board. DPV was performed at a pulse amplitude of 50 mV, a pulse width of 70 ms and a scan rate of 5 mV s^–1^.

The dotted line in Fig. 9 shows the DPV obtained in the absence of carboplatin (0 μM). The decrease in the AMP oxidation peak current corresponding to an external beaker concentration of 10 μM was clearly observed (representing an on-chip concentration of less than this due to microdialysis probe recovery being less than 100%). The detection limit under these conditions is 0.014 μM, lower than with on-chip calibration. The reasons for this improvement are not clear. This experiment is a proof of principle of the carboplatin detection method.

## 4 Conclusions

The fabricated microfluidic platform coupled with an MD probe was successfully used to detect the interaction of carboplatin and AMP on-line. A decrease in the oxidation current of AMP was clearly observed when the dialysate containing carboplatin interacted with the AMP solution on the microfluidic board. We have chosen AMP as it shows greatest sensitivity to carboplatin. The detection limit of our device is in the appropriate range for *in vivo* carboplatin cytotoxicity measurements. This fabricated system has potential for development of a clinical device for the detection of carboplatin in real time during chemotherapy.

## Author contributions

TP carried out the experiments, wrote and revised the manuscript, CLL assisted with experiment and revised the manuscript, SG assisted with experiments and revised the manuscript, BAP assisted with experiments and revised the manuscript, MGB directed the experiments, wrote and revised the manuscript.
