# Supplementary material for: Real-time detection of carboplatin using a microfluidic system
Source: Analyst. 2016 Aug 26;141(22):6270–7. doi: 10.1039/c6an01446a (PMC5123639; doi:10.1039/c6an01446a)
Supplement: Supplementary file 1 [file AN-141-C6AN01446A-s001.pdf]

Electronic Supplementary Material (ESI) for Analyst.
This journal is © The Royal Society of Chemistry 2016

Electronic Supplementary Information for:

Real-time detection of carboplatin using a microfluidic system†

Tonghathai Phairatana,[a,b] Chi Leng Leong,[b] Sally A N Gowers,[b] Bhavik Anil Patel,[c] and Martyn G Boutelle*[b]

Optimisation of the Analysis Protocol

The process of cleaning the electrode and detecting carboplatin was very time-consuming (each scan was in the range from 0 V to 1.6 V at a scan rate of 5 mV/s). It is a challenging task to reduce this time so that detection of carboplatin is as short as possible making it more suitable for real-time clinical use.

1. **Detection of Carboplatin Using a MWCNT-epoxy Composite Electrode**

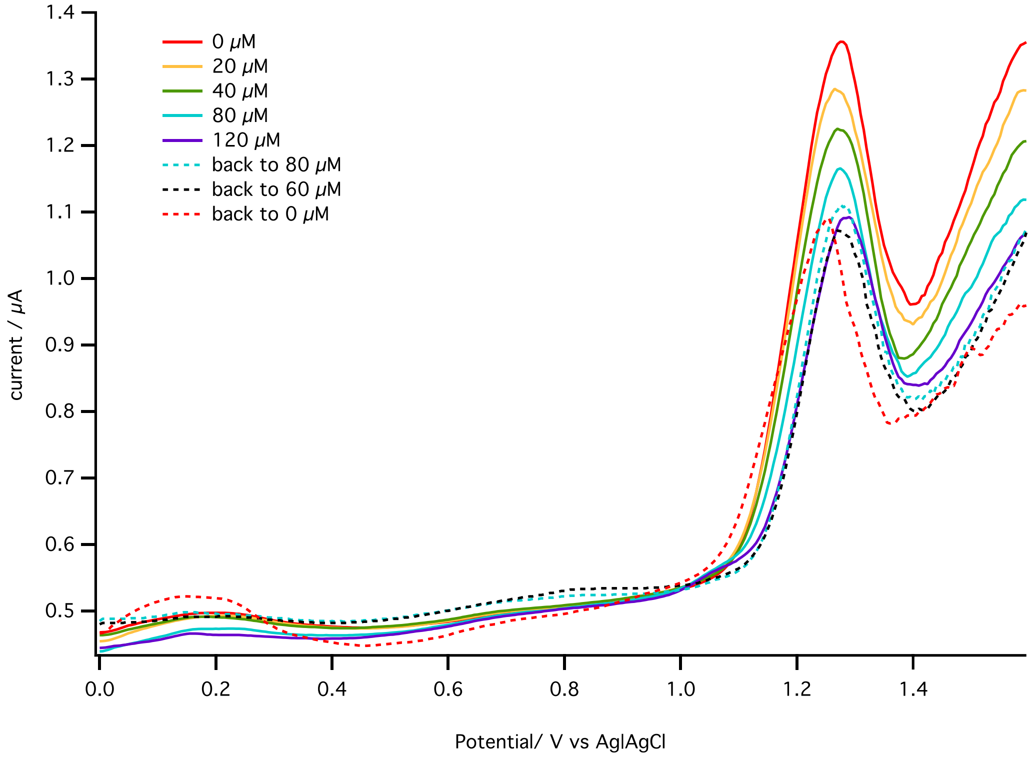

Figure S1: DPVgrams of a bare MWCNT-epoxy composite sensor for the addition of 0 to 120 µM carboplatin to 20 ml of 1.5 mM AMP in ACB/T1 in 1.5:0.5 ratio (AMP/ACB/T1) solution under stirred conditions. The dotted lines show the results after addition of 1.5 mM AMP/ACB/T1 solution to dilute the carboplatin concentration in the beaker. Pulse amplitude was 50 mV, pulse width was 70 ms and scan rate was 5 mV/s.

We can see that as expected carboplatin reduces the height of the AMP oxidation peak in a dose-dependent manner. However, this effect seems to be irreversible. When 1.5 mM AMP in ACB/T1 (1.5:0.5 AMP/ACB/T1) solution was added into the beaker to dilute the final carboplatin concentration (from 80 µM to 60 µM), the oxidation peak did not recover to the expected current. Furthermore, when the MWCNT-epoxy composite electrode was tested in fresh 1.5 mM AMP/ACB/T1 solution (0 µM carboplatin), the oxidation current observed did not

recover to its pre-testing value. The fact that the current does not increase again in a fresh carboplatin-free solution strongly suggests that the effect is due to a change on the electrode surface. To examine the causes of this surprising result, a stepwise protocol was tested.

1.2 **Electrochemical Cleaning of MWCNT-epoxy Composite Electrode**

Inhibition of the AMP oxidation peak occurred following oxidation of AMP in the presence of carboplatin. To investigate whether applying a negative potential could be used to clean the electrode surface after carboplatin detection, the MWCNT-epoxy composite electrode was held at different constant holding potentials (-1 V, -2 V, -0.5 V, -0.2 V and -0.1 V vs Ag|AgCl) in 1.5 mM AMP/ACB/T1 solution in the absence of carboplatin for 2 minutes.

For each different holding potential the electrode was first placed in a beaker containing 40 μM carboplatin in 1.5 mM AMP/ACB/T1 solution and DPV was performed in the range from 0 V to 1.6 V to demonstrate peak inhibition. Following this, the working electrode was washed with ACB to ensure no carboplatin solution remained on the electrode surface. The electrode was then moved to a beaker containing 1.5 mM AMP/ACB/T1 (0 μM carboplatin) and held at the chosen holding potential for 2 minutes after which DPV was performed. Figure S2 illustrates the results.

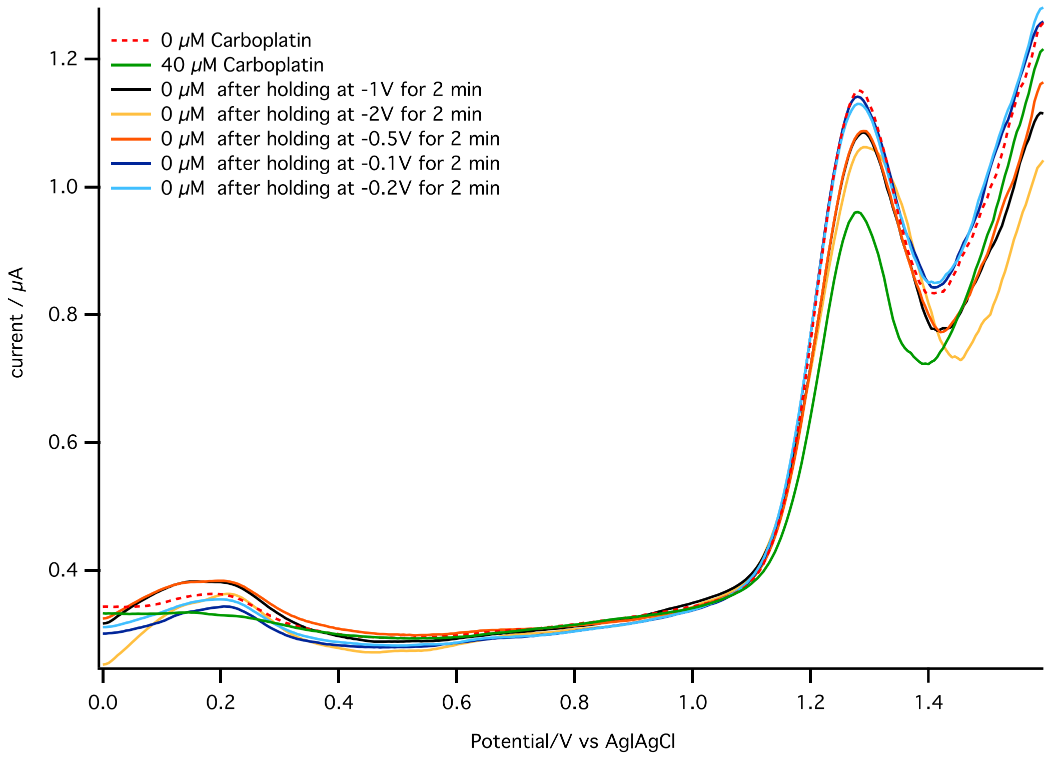

Figure S2: DPVgrams obtained after holding the electrode at different constant potentials to clean the electrode surface. These potentials include -1 V, -2 V, -0.5 V, -0.1 V and -0.2 V. DPV was performed in a beaker with a pulse amplitude of 50 mV, a pulse width of 70 ms, and a scan rate of 5 mV/s.

As seen in Figure S2, the oxidation current recovered to close to its initial value after holding the electrode at a constant potential of -0.1 V (dark blue trace). Thus, the constant holding potential of -0.1 V was chosen for further experiments to clean the electrode surface after carboplatin detection in a microfluidic platform.

Static Holding Times

The time that the electrode was held at -0.1 V and then at 0 V was varied to ascertain the minimum length of time required at each stage. For each condition the experiment was conducted following this procedure:

1. The electrode was placed in a beaker containing 1.5 mM AMP/ACB/T1 solution in the absence of carboplatin (0 µM carboplatin) and held at 0 V for 2 minutes. DPV was performed from 0-1.5 V (shown as a black trace in Figure S3).
2. The electrode was placed in a beaker containing 40 µM carboplatin in 1.5 mM AMP/ACB/T1 and DPV was performed from 0-1.5 V (shown as a green trace in Figure S3).
3. The electrode was washed with ACB to ensure no carboplatin solution

remained on the electrode surface.
4. The electrode was cleaned in 0 μM carboplatin by holding it at -0.1 V for the required time.
5. The background current was allowed to stabilise at 0 V for the required time and DPV was performed.
6. Steps 2-5 were repeated.

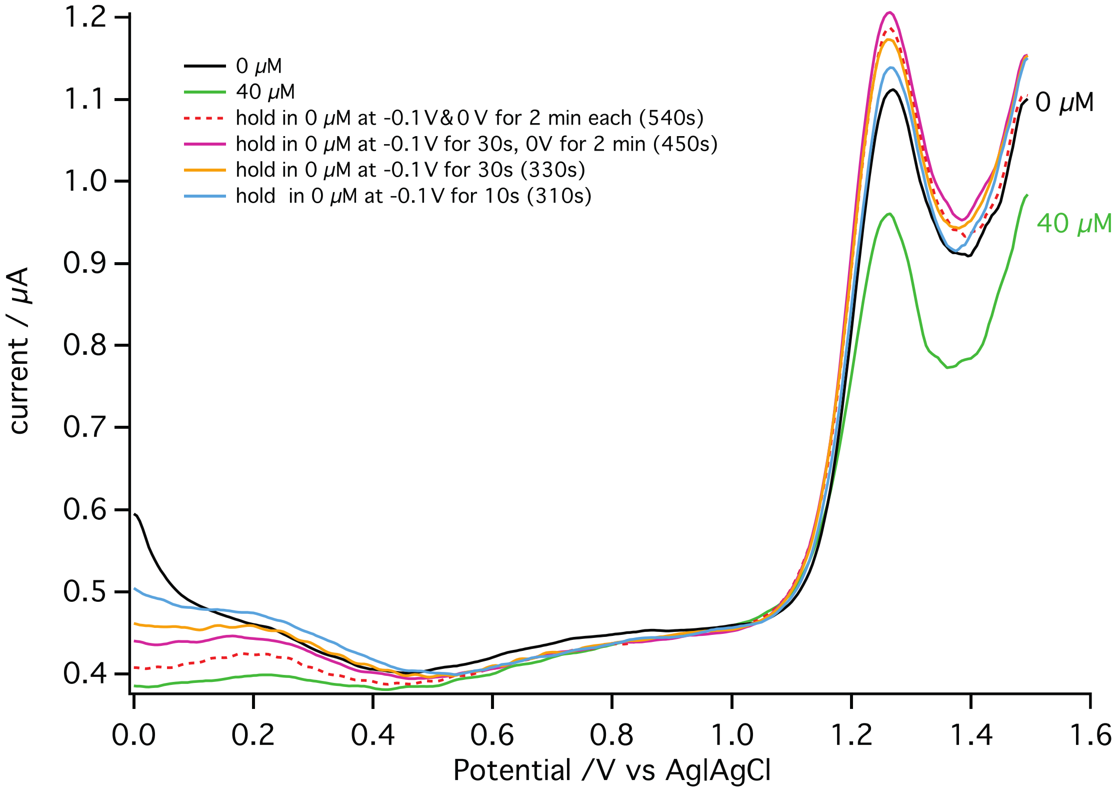

Figure S3: DPVgrams obtained showing the effect of holding the electrode at -0.1 V for different lengths of time (2 minutes, 30 s and 10 s) for the cleaning step and with/without holding the electrode at 0 V for 2 minutes to allow the baseline to stabilise. DPV was performed (0 V-1.5 V) in a beaker under stirred conditions with a pulse amplitude of 50 mV, a pulse width of 70 ms and a scan rate of 5 mV/s. The black trace shows the initial AMP oxidation peak before testing with carboplatin and the green trace shows the AMP oxidation peak in 40 μM carboplatin in 1.5 mM AMP/ACB/T1.

Figure S3 shows the results of investigating holding the electrode at -0.1 V for 2 minutes, 30 s and 10 s for the cleaning step and of whether it is necessary to hold the electrode at 0 V for 2 minutes to allow the baseline to stabilise. In all cases, the DPVgrams showed enhancement of the sensitivity beyond that of the initial DPV scan (black trace) after holding the electrode at -0.1 V in 1.5 mM AMP/ACB/T1. It can also be seen that it was not necessary to hold the electrode at 0 V for 2 minutes for baseline stabilisation. The electrode could be held at -0.1 V for 10 s, but there was a slight improvement in the sensitivity when this was increased to 30 s. From this result, we can conclude that the stabilisation step at 0 V is not required and the cleaning step at -0.1 V could be reduced to 30 s.

Therefore, these steps were reduced from 4 minutes to only 30 s in total, leading to a total reduction in the procedure length of 330 s.

DPV Scan Time

Next, a study to investigate what range of potentials should be scanned was carried out. The scan range for DPV was performed from 0.8 V to 1.5 V instead of from 0 to 1.6 V at a scan rate of 5 mV/s. The results are shown in Figure S4.

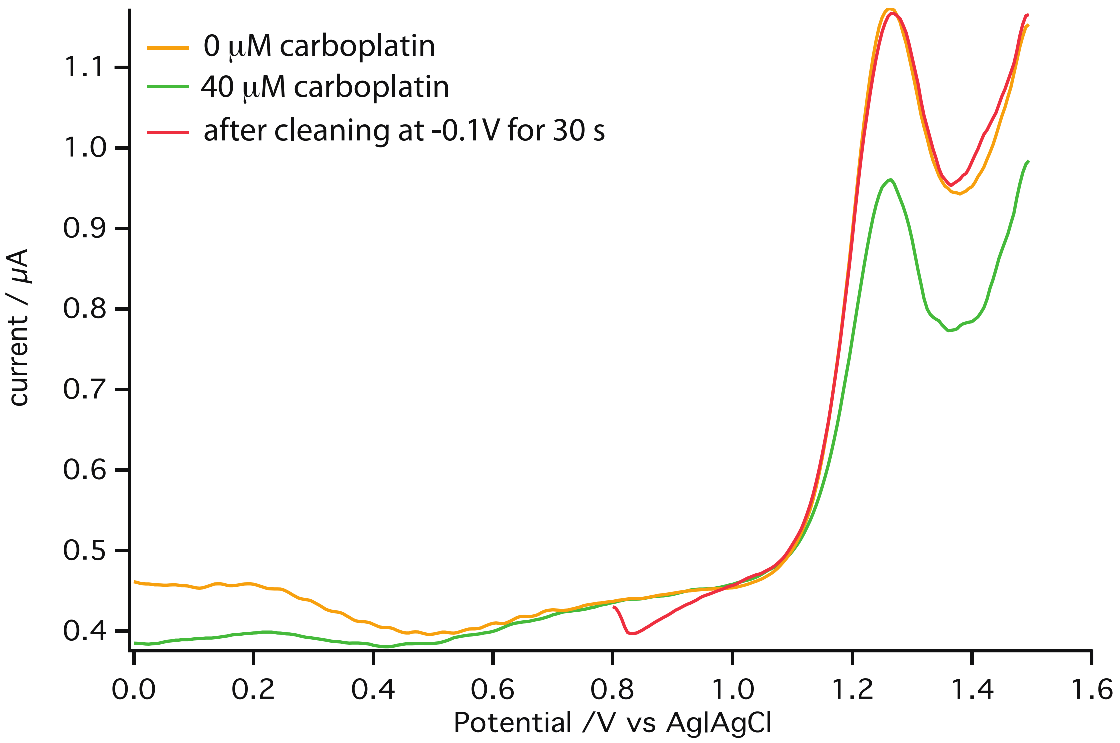

Figure S4: DPVgrams obtained before and after carboplatin detection and after the electrode is held at -0.1 V for 30 s. The potential range scanned is either 0-1.5 V or 0.8-1.5 V. DPV was performed in a beaker under stirred conditions with a pulse amplitude of 50 mV, a pulse width of 70 ms and a scan rate of 5 mV/s.

Peak recovery after carboplatin detection was obtained even when the scan potential range was reduced from 0-1.6 V to 0.8-1.5 V as shown in Figure S4. For the shorter scan range, the potential jumped from -0.1 V to 0.8 V at the beginning of the scan. Indeed 0.9 V was found to be too high to use as the DPV starting potential as this caused a potentiostatic step to interfere with the baseline at around 1.0 V, making it very difficult to measure the DPV oxidation peak (data not shown). After holding the electrode at -0.1 V for 30 s, the effect of carboplatin on the AMP oxidation peak was reversed and the current peak was equivalent to that for when the full range was scanned (yellow trace).

A summary of the conditions chosen to minimise the length of time

required for carboplatin detection at MWCNT-epoxy composite electrodes and to clean the electrodes is given in Table S1.

Table S1: Summary of the conditions chosen for detection of carboplatin at MWCNT-epoxy composite electrodes.

**Conditions chosen**	
Scan potential range	0.8-1.5 V
Holding potential	-0.1 V
Time-period for holding potential	30 s
